# MORG1—A Negative Modulator of Renal Lipid Metabolism in Murine Diabetes

**DOI:** 10.3390/biomedicines10010030

**Published:** 2021-12-23

**Authors:** Eric Jankowski, Sophie Wulf, Nadja Ziller, Gunter Wolf, Ivonne Loeffler

**Affiliations:** Department of Internal Medicine III, Jena University Hospital, Am Klinikum 1, D-07747 Jena, Germany; Eric.Jankowski@med.uni-jena.de (E.J.); Sophie.Wulf@med.uni-jena.de (S.W.); Nadja.ziller@med.uni-jena.de (N.Z.); gunter.wolf@med.uni-jena.de (G.W.)

**Keywords:** type 1 diabetes mellitus, T1DM, type 2 diabetes mellitus, T2DM, diabetic nephropathy, kidney, lipid metabolism, fatty acid metabolism, MORG1, mitogen-activated protein kinase organizer 1, WDR83

## Abstract

Renal fatty acid (FA) metabolism is severely altered in type 1 and 2 diabetes mellitus (T1DM and T2DM). Increasing evidence suggests that altered lipid metabolism is linked to tubulointerstitial fibrosis (TIF). Our previous work has demonstrated that mice with reduced MORG1 expression, a scaffold protein in HIF and ERK signaling, are protected against TIF in the db/db mouse model. Renal TGF-ß1 expression and EMT-like changes were reduced in mice with single-allele deficiency of MORG1. Given the well-known role of HIF and ERK signaling in metabolic regulation, here we examined whether protection was also associated with a restoration of lipid metabolism. Despite similar features of TIF in T1DM and T2DM, diabetes-associated changes in renal lipid metabolism differ between both diseases. We found that de novo synthesis of FA/cholesterol and β-oxidation were more strongly disrupted in T1DM, whereas pathological fat uptake into tubular cells mediates lipotoxicity in T2DM. Thus, diminished MORG1 expression exerts renoprotection in the diabetic nephropathy by modulating important factors of TIF and lipid dysregulation to a variable extent in T1DM and T2DM. Prospectively, targeting MORG1 appears to be a promising strategy to reduce lipid metabolic alterations in diabetic nephropathy.

## 1. Introduction

One of the most important complications of type 1 (T1DM) and type 2 diabetes mellitus (T2DM) is diabetic nephropathy (DN) [1,2]. Early in DN, the interstitium becomes dilated, leading to tubulointerstitial fibrosis (TIF) and tubular atrophy [3,4]. In TIF, myofibroblast accumulation is observed, accompanied by excessive extracellular matrix (ECM) deposition and, in later stages, destruction of renal tubules [5,6]. Fibrosis-promoting cytokines (e.g., transforming growth factor beta 1 (TGF-β1), connective tissue growth factor (CTGF)) are produced in fibrogenic signaling phase of TIF [7]. In contemporary understanding of renal fibrosis, multiple cell types and different mechanisms appear to be responsible for ECM accumulation and remodeling [8,9,10,11,12,13,14,15]. Recently, it has been demonstrated that the downregulation of key enzymes and regulators of fatty acid oxidation (FAO), as well as increased lipid accumulation in proximal tubular epithelial cells, is directly linked to TIF [16]. Under physiological conditions, tubular cells rely on fatty acids (FAs) as their main energy source. Therefore, uptake of FAs, FA oxidation and FA synthesis are tightly balanced in these cells [16,17,18,19]. Conversely, injured tubular cells display dramatic metabolic rearrangements, including profound suppression of FAO [17]. This altered renal lipid metabolism has been described in DN, where sustained hyperglycemia promotes FA synthesis and triglyceride accumulation [20]. The elevated serum triglycerides, together with free FAs and modified cholesterol, can cause lipid accumulation in non-adipose tissues, a process termed lipotoxicity [20].

Various pathological stimuli in the diabetic milieu, such as TGF-β1 and hypoxia, are known to induce an imbalance in lipid metabolism [21,22,23]. Tissue hypoxia in patients with chronic kidney disease results from an altered renal oxygen supply, thus leading to the stabilization of the hypoxia-dependent transcription factors HIF1/2 (hypoxia-inducible factor 1/2) in the kidneys [24]. HIF1α and HIF2α function mainly as transcriptional activators, regulating several biological processes, such as metabolism of glucose, cholesterol and FAs [25,26,27]. In contrast to the apparent role of HIF in carbohydrate metabolism, its effects on lipid metabolism have recently been investigated [21]. In addition to the HIF pathway, the MEK/ERK pathway also appears to be involved in lipid metabolism, as extracellular signal-regulated kinases 1/2 (ERK1/2) can influence several master regulators of cellular lipid metabolism [28,29,30,31].

MORG1 (mitogen-activated protein kinase organizer 1, also known as WDR83) is identified as a scaffold protein in both ERK1/2 and HIF pathways. It is a member of the WD-40 domain protein family with a molecular mass of 34.5 kDa and is ubiquitously expressed in different organs, including the heart, brain and kidney [32]. MORG1 was first isolated as a binding partner of the ERK pathway scaffold protein MP1 (MEK partner 1) in 2004 [32]. It also specifically associates with multiple-components of the mitogen-activated protein kinase (MAPK) cascade and stabilizes their assembly into an oligomeric complex [32]. Furthermore, MORG1 biphasically modulates the activation of the ERK cascade: at low concentrations, MORG1 enhances ERK activation, whereas high concentrations lead rather to the inhibition of ERK activation [32]. In addition to its central role in the MAPK pathway, MORG1 also functions as a scaffold protein in HIF signaling. Our group has identified MORG1 as a scaffold protein of PHD3 (prolyl hydroxylase domain protein 3) that regulates the degradation of HIF-1α and HIF-2α under normoxic conditions [24,33]. MORG1 was found to interact with PHD3, leading to the stabilization of PHD3. In this way, MORG1 works together with PHD3 in the regulation and degradation of HIF-α protein [33,34,35]. By suppression of MORG1 the basal HIF-α protein stability is increased and (as a result) there is reduction in HIF-α degradation [33,36,37,38]. Several lines of evidence support the notion that MORG1 is involved in the pathophysiology of various diseases. Due to embryonic lethality of homozygous MORG1 knockout mice, studies have been conducted in heterozygous mice that exhibit a normal phenotype. We described that heterozygous MORG1 knockout mice are protected from experimentally induced focal cerebral ischemia [39], from acute renal ischemia-reperfusion injury [40] and from acute kidney damage due to systemic hypoxia [37]. Recent data indicate that they are also protected from kidney damage as a late consequence of T2DM [41]. In the db/db mouse model for type 2 DN, the DN was ameliorated when MORG1 expression was suppressed [41]. We hypothesize that the attenuated TIF is, at least partly, a consequence of reduced EMT (epithelial-to-mesenchymal transition)-like changes in tubular cells [41].

In this study, we aimed to investigate whether a reduction of MORG1 in kidneys of diabetic mice leads to a restoration of a potentially disrupted lipid metabolism. Here, we compare two different diabetes models: insulin-dependent (STZ-induced T1DM-like phenotype) and insulin-independent diabetes mellitus (db/db mouse model for T2DM).

## 2. Materials and Methods

### 2.1. Animals

All animal experiments were approved by the Local Ethics Committee of ThüringerLandesamt für Verbraucherschutz (approval numbers UKJ-17-024 and 02-039/15) and were performed in accordance with the German Animal Protection Law. The animals were housed in a pathogen-free facility with a 12-h light–dark cycle and raised on standard chow and water ad libitum. MORG1 knockout mice were derived from the C57BL6/J strain and generated by homologous recombination, using standard techniques [42]. A cassette containing various selection markers and the gene for green fluorescence protein was placed in the exons 1–5 of the MORG1 gene to disrupt the MORG1 coding sequence and to detect the expression. Individuals involved in this study were backcrossed to the C57BLKS background for at least 10 generations. MORG1^+/−^ (heterozygous) mice exhibited a normal phenotype; however, MORG1^−/−^ (homozygous) animals exhibited embryonal lethality between embryonic days 8.5 and 10.5, probably due to diffuse vascularization of the embryo and malformations in the neural tube (unpublished observations).

Mouse model for T1DM-like phenotype: Mild insulin-dependent diabetes mellitus was induced in wild-type (*n* = 7) and MORG1 heterozygous mice (*n* = 7) at 12–15 weeks of age by intraperitoneal administration of 50 mg/kg streptozotocin (STZ; Sigma-Aldrich, St. Louis, MO, USA; in sterile 10 mM sodium citrate, pH 5.5) for 4 consecutive days. Due to their genetic background, which contains proportions of DBA/2, the animals used were very good STZ responders [43]. Therefore, the used STZ protocol was sufficient to keep fasting blood glucose stable above 15 mmol/L (on average) and to avoid ketonuria. Non-diabetic control animals received daily intraperitoneal injections of placebo (10 mM sodium citrate, pH 5.5) for 4 days. After 3 months of inducing diabetes, the mice were euthanized and their kidneys were removed. One half of the kidney was fixed in 4% phosphate buffered formalin and for histological studies embedded in paraffin. Remaining tissue was snap-frozen for mRNA analysis.

T2DM mouse model: The mouse line used for our T2DM model was generated by crossing mice from the db/db strain (BKS.Cg-Dock7m+/+Leprdb/J; C57BLKS/J background; obtained from Charles River Laboratory (Brussels, Belgium)) with mice from the MORG1 strain. Based on their genotype, the animals were divided into four groups: MORG1 wild-type non-diabetic control (db/m; MORG1^+/+^) (*n* = 10), MORG1 wild-type diabetic T2DM (db/db; MORG1^+/+^) (*n* = 8), MORG1 heterozygous non-diabetic control (db/m; MORG1^+/−^) (*n* = 5) and MORG1 heterozygous diabetic T2DM (db/db; MORG1^+/−^) (*n* = 6). At the age of 25–28 weeks, mice were euthanized, and their kidneys were removed. One half of a kidney was fixed in 4% phosphate buffered formalin and for histological studies embedded in paraffin. Remaining tissue was snap-frozen for mRNA analysis and Oil Red O staining.

### 2.2. Immunohistochemistry and Oil Red O Staining

In preparation for immunohistochemical staining, the 3 µm paraffin sections were dewaxed/deparaffinized and rehydrated. A heat-mediated antigen retrieval procedure in citrate buffer (pH 6.0) was performed. Blocking of endogenous peroxidase was achieved by incubation with 3% H2O2 (Roth, Karlsruhe, Germany) for 10 min at room temperature. Following the blocking with Roti-Block, the sections were incubated with primary antibodies overnight at 4 °C. The following primary antibodies were used: rabbit polyclonal anti-fatty acid synthase (FAS) antibody (Abcam, Camebridge, UK), mouse monoclonal anti-MTCO1 antibody (Abcam, Camebridge, UK) and rabbit polyclonal anti-PPARαantibody (Abcam, Cambridge, UK). After incubation with peroxidase-labeled goat anti-rabbit IgG antibody (KPL, Gaithersburg, MD, USA) or anti-mouse IgG antibody (SeraCare, Milford, MA, USA), di-aminobenzidine (DAB) (DAB-peroxidase substrate kit; Vector Laboratories, Burlingame, CA, USA) was used as a chromogen.

For Oil Red O staining, snap-frozen renal sections (thickness 10 µm) were stained by using Oil Red O solution 0.5% in iso-propanol (Sigma-Aldrich, Merck, Darmstadt, Germany).

### 2.3. Immunofluorescence

Protein expression via immunofluorescence was analyzed on paraffin-embedded kidney sections (thickness 3 µm). After deparaffination, hydration and heat-mediated antigen retrieval in citrate buffer (pH 6.0), blocking with 5% BSA (bovine serum albumin, Roth, Karlsruhe, Germany) was performed for one hour at room temperature. The kidney sections were incubated with recombinant anti-CD36 antibody (Abcam, Camebridge, UK) at 4 °C overnight. As secondary antibody anti-rabbit IgG DY-light 594 (Vector Laboratories, Burlingame, CA, USA) was used. Nuclei were counterstained with 4′,6-diamidino-2-phenylindole (DAPI, Sigma-Aldrich, Merck, Darmstadt, Germany).

### 2.4. Quantification

For quantification, at least 10 non-overlapping high-power fields (magnification ×200) for each kidney sample were examined by AxioVision 4.8 software monochrome modus of the AxioCamHRc camera (both Zeiss, Jena, Germany) and analyzed by using an ImageJ Macro. ZeissVisionImage (.zvi) format was exported to TIFF format. Gained data were analyzed by using the thresholding function of ImageJ. For each staining, an individual threshold was customized and applied to all images of that staining. Staining was segmented into relevant and irrelevant. As an example, the used MACRO for FAS staining can be found in the Supplementary Materials and Section 2.

Obtained data of relevantly stained area per image were averaged for each mouse. To compare individuals of different groups, all mice were normalized to the mean value of samples from the MORG1 wild-type non-diabetic control group (db/m; MORG1^+/+^).

### 2.5. cDNA Synthesis and Semi-Quantitative Real-Time PCR

For isolation of total RNA from kidney cortex, the NucleoSpin 8 RNA Kit (Macherey-Nagel, Düren, Germany) was used. Elimination of possible DNA contamination was performed with the RNase-Free DNase Set (Qiagen, Hilden, Germany), and 1 µg total RNA was reverse-transcripted into cDNA with the Reverse-Transcription System Promega (Promega, Madison, WI, USA). Determination of gene-expression levels was performed by semi-quantitative real-time PCR by use of the LightCycler-FastStart DNA Master SYBR Green 1 (Roche Diagnostics, Mannheim, Germany) and a thermocycler (qTower, Analytik Jena, Jena, Germany). PCRs were carried out with sense and antisense primers at a concentration of 0.25 µM each (purchased from TIB Molbiol, Berlin, Germany). Temperatures and sequences of all primer pairs are shown in Table 1. Hypoxanthine phospho-ribosyltransferase 1 (HPRT1) was used as the housekeeping gene. Relative expression ratio was quantified by the ΔΔCT method, and transcript levels were normalized to the mean value of the MORG1 wild-type non-diabetic control group.

### 2.6. Statistics

The data from the four experimental groups are shown as box/whisker-dot plots, drawn using SPSS statistics (IBM company, Armonk, NY, USA). The boxes’ boundaries mark the 25th percentile in the bottom and the 75th percentile on the top. The line in the center of the box indicates the median of values and the whiskers, drawn below and above the box, span from the 10th to the 90th percentile. Values with a distance of ≥1.5*IQR (interquartile range) to the first and third quartile are defined as outliers. Outliers (presented as single dots outside the whiskers) were excluded from statistical analysis. Effects (main effects and interactions) were assessed by using two-way analysis of variance (ANOVA) with phenotype (non-diabetic control or diabetic (STZ-T1DM/T2DM)) and genotype (wild-type or heterozygous) as the two factors. Interaction determines whether the one main effect depends on the level of the other main effect. For intergroup comparison, Mann–Whitney U test was used to test the statistically significance between two independent groups (MORG1 wild-type control vs. MORG1 wild-type STZ-T1DM/T2DM; MORG1 wild-type control vs. MORG1 heterozygous control; MORG1 heterozygous control vs. MORG1 heterozygous STZ-T1DM/T2DM; MORG1 wild-type STZ-T1DM/T2DM vs. MORG1 heterozygous STZ-T1DM/T2DM).

To analyze the correlation between tested lipid metabolism markers and CTGF, KIM1 or lipid accumulation, all groups were included, and the Pearson correlation coefficient (ρ) was calculated by using SPSS statistics. To analyze the correlation between PPARα and FAS or CD36 expression, only non-diabetic mice were included, and the Pearson correlation coefficient (ρ) was calculated. We classified Pearson’s correlations as reasonable according to medical standards, with ρ of 0.3 to 0.5 as fair, 0.6 to 0.7 as moderate and >0.7 as very strong [52].

A *p*-value of ≤0.05 was considered statistically significant. The *p*-values from ≥0.05 to ≤0.1 were considered biologically relevant.

## 3. Results and Discussion

### 3.1. Different Role of MORG1 in Renal Fatty Acid and Cholesterol Synthesis in STZ-T1DM and T2DM

A tubule epithelial lipid accumulation was found in patients with manifested DN. Lipid droplets are round membrane-coated organelles, which contain potentially toxic triglycerides and cholesterol esters [20]. It is believed that genes involved in triglyceride, as well as cholesterol, metabolism are dysregulated in DN. In diabetic kidneys, increased uptake of FAs, de novo synthesis of FAs and triglycerides and decreased expression of master regulators of FAO may contribute to lipid storage, thus contributing to the altered expression of receptors or transporters regulating the influx/efflux of cholesterol [16,17,19,20].

**Figure 1:**
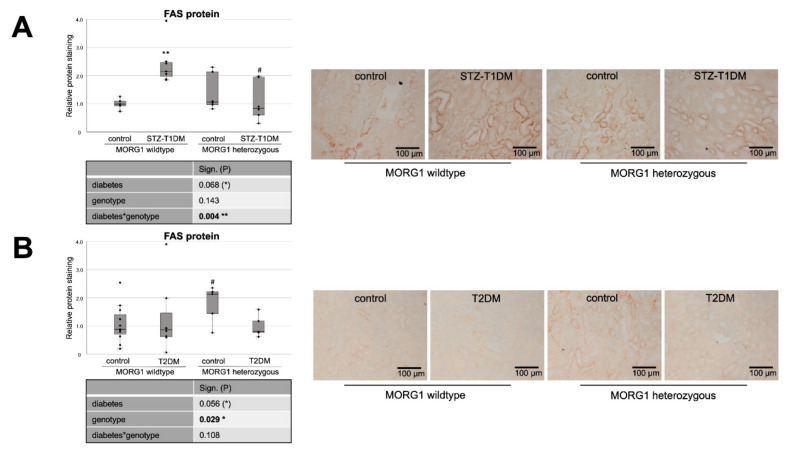
Role of MORG1 in de novo synthesis of fatty acids in tubular cells. (**A**,**B**) Immunohistochemistry of FAS (fatty acid synthase) in STZ-T1DM and T2DM and semi-quantitative analysis of the staining. Representative images are shown next to the graphs (magnification ×200). The significances shown in the tables were assessed by two-way ANOVA with diabetes (control or STZ-T1DM/T2DM) and genotype (MORG1 wild-type or MORG1 heterozygous) as the two factors. Diabetes, genotype: main effect of diabetes/genotype, respectively. Diabetes*genotype: interaction. Bold: statistically significant. (*) Biologically relevant. Significances shown next to each bar were assessed by using Mann–Whitney U test; ** *p* ≤ 0.01 in between same genotype; # *p* ≤ 0.05 in between different genotypes.

**Figure 2:**
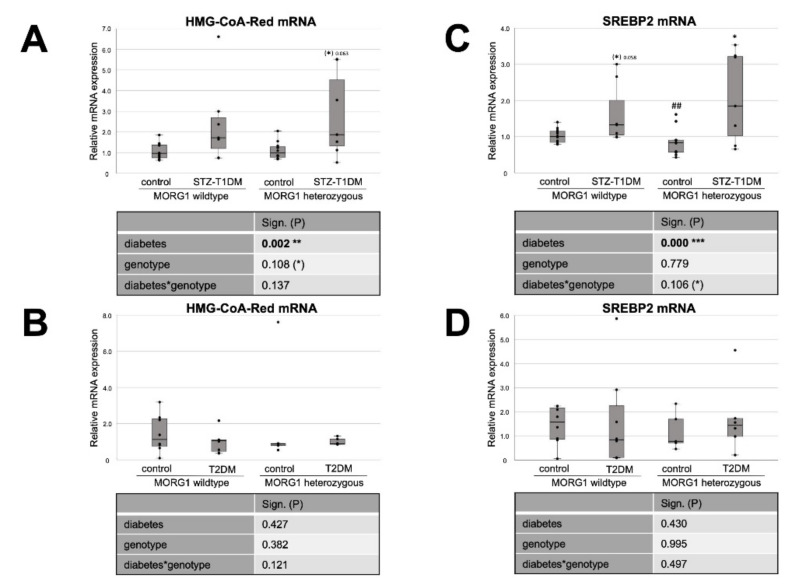
Effect of MORG1 and diabetes on renal expression of key enzymes of cholesterol synthesis. (**A**,**B**) Real-time analysis of HMG-CoA-Red (3-hydroxy-3-methylglutaryl-CoA-reductase) mRNA expression in STZ-T1DM and T2DM. (**C**,**D**) Real-time mRNA analysis of SREBP2 (sterol regulatory element-binding protein 2) in STZ-T1DM and T2DM. (**A**–**D**) The significances shown in the tables were assessed by two-way ANOVA with diabetes (control or STZ-T1DM/T2DM) and genotype (MORG1 wild-type or MORG1 heterozygous) as the two factors. Diabetes, genotype: main effect of diabetes/genotype, respectively. Diabetes*genotype: interaction. Bold: statistically significant. (*) Biologically relevant. Significances shown next to each bar were assessed by using Mann–Whitney U test; * *p* ≤ 0.05 in between same genotype; ## *p* ≤ 0.01 in between different genotypes; (*) biological relevant in between same genotypes.

Firstly, we investigated the expression of markers for FA and cholesterol synthesis in our models. Sterol regulatory element binding proteins (SREBPs) serve as the master regulators of cellular FA and cholesterol synthesis, whereas SREBP-2 regulates cholesterol synthesis. FA synthesis is regulated by SREBP-1 and catalyzed through FA synthase (FAS) and acetyl-CoA carboxylase (ACC) [20,21]. Our analysis revealed differences not only between the types of diabetes, but also regarding the MORG1 genotype in this condition (Figure 1 and Figure 2). In mice with T1DM-like phenotype, FAS protein, as well as HMG-CoA-Red and SREBP2 mRNA, was upregulated (Figure 1A and Figure 2A,C), and this correlates with previous data [20]. Whereas the single-allele deficiency of MORG1 resulted in the restoration of diabetes-induced FAS dysregulation (Figure 1A), the upregulation of cholesterol synthesis was independent of MORG1 genotype (Figure 2C). Analysis of variance (represented in tables below the graphs) confirms a significant diabetes effect on FA and cholesterol synthesis (Figure 1A and Figure 2A,C) and a profound interaction between diabetes phenotype and MORG1 genotype on protein expression of FAS (Figure 1A).

The similar analysis of kidneys from T2DM mice (Figure 1B and Figure 2B,D) did not yield significant results, except that the non-diabetic control animals with MORG1 heterozygous genotype showed significantly higher FAS expression compared to the wild-type control (Figure 1B). This could also be observed in the non-diabetic animals of the STZ-T1DM model (Figure 1A). A possible explanation is the HIF1/2 stabilization in MORG1 heterozygous mice, which was shown in previous work [37,41]. An HIF1-dependent activation of SREBP1 and, thus, increased expression of FAS have been described [21].

Studies of a possible diabetes effects on renal MORG1 expression revealed that neither T2DM nor STZ-induced diabetes affected tubular MORG1 protein expression in our models. Thus, renal MORG1 levels are similar in non-diabetic and diabetic kidneys, with an overall reduction of MORG1 of approximately 20–25% in heterozygous mice (Supplementary Materials Figure S1 and Reference [41]).

In summary, we demonstrated that there is a significant interaction between MORG1 expression and STZ-T1DM in the synthesis of FA. The results further suggest that, in our T2DM model, increased FA or cholesterol synthesis does not play a role in lipid accumulation. On the contrary and consistent with the trend observed in wild-type animals with T2DM (Figure 2D), gene expression analyses in kidney biopsies of patients with DN (with T2DM) showed decreased FAS, as well as SREBP2 [20].

### 3.2. Expression of Fat Transporters in Tubular Cells Depends on Type of Diabetes and MORG1 Level

Intracellular lipid accumulation can result from de novo synthesis of FAs and cholesterol or from exogenous FAs uptake and cholesterol influx. CD36 (cluster of differentiation 36) is a scavenger receptor class B that mediates binding and uptake of long-chain FAs, oxidized lipids and phospholipids, advanced oxidation protein products, advanced glycation end-products (AGEs) and thrombospondin [53]. In the kidney, CD36 is mainly expressed in tubular epithelial cells, podocytes and mesangial cells, and multiple ligands regulate its expression and intracellular location [53]. Moreover, we have demonstrated for the first time that the scavenger receptor class B type I, a specific receptor for HDL is mainly expressed in proximal tubular cells and is downregulated by angiotensin II (ANG II) [54]. Since local ANG II concentrations are high in DN [2], this mechanism may contribute to severe lipid abnormalities in DN.

**Figure 3:**
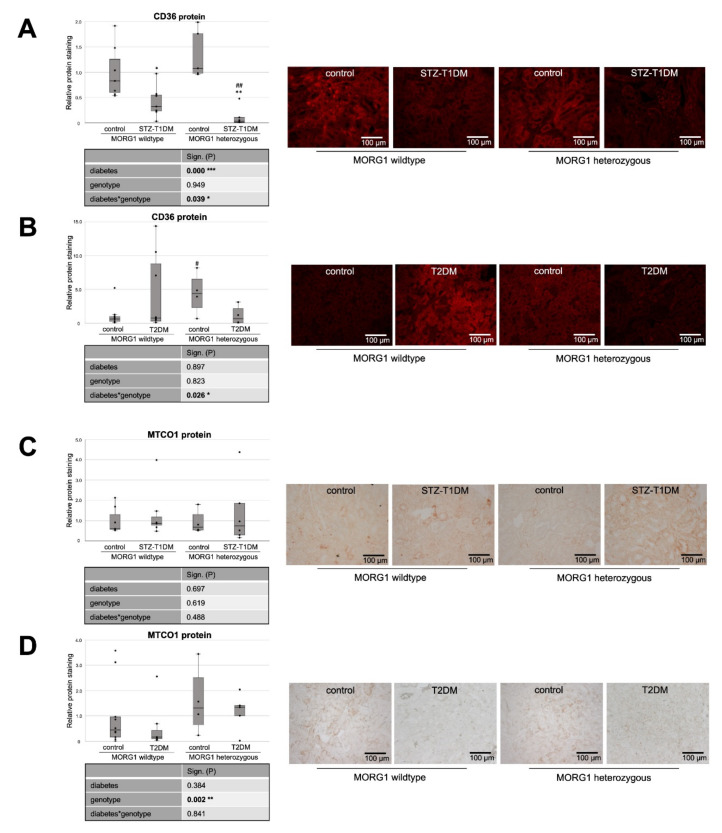
Analysis of fat uptake and metabolic stress in kidneys of diabetic animals. (**A**,**B**) Immunofluorescence staining of CD36 (cluster of differentiation 36) and semi-quantitative analysis of the staining in STZ-T1DM and T2DM. (**C**,**D**) Immunohistochemistry of MTCO1 (mitochondrially encoded cytochrome C oxidase 1) in STZ-T1DM and T2DM and semi-quantitative analysis of the staining. (**A**–**D**) Representative images are shown next to the graph (magnification ×200). The significances shown in the tables were assessed by two-way ANOVA with diabetes (control or STZ-T1DM/T2DM) and genotype (MORG1 wild-type or MORG1 heterozygous) as the two factors. Diabetes, genotype: main effect of diabetes/genotype, respectively. Diabetes*genotype: interaction. Bold: statistically significant. Significances shown next to each bar were assessed by using Mann–Whitney U test; * *p* ≤ 0.05 in between same genotype; ** *p* ≤ 0.01 in between same genotype; # *p* ≤ 0.05 in between different genotypes; ## *p* ≤ 0.01 in between different genotypes.

Experimental induction of a T1DM-like phenotype significantly reduces tubular CD36 protein in our model, which is further decreased by reduction of MORG1 (Figure 3A). Variance analysis reveals significant diabetes–MORG1 interaction. The data suggest that, in this diabetes model, increased de novo synthesis, but not increased uptake, contributes to impaired lipid metabolism.

However, a somewhat different situation emerges in the T2DM model (Figure 3B). Although not statistically significant across all animals tested in the intergroup comparison, we found accelerated CD36 expression in the kidneys of some mice with T2DM (Figure 3B). This is consistent with the finding that patients with diagnosed type 2 DN showed increased fat uptake receptor expression in kidneys (e.g., CD36) [20].

HK-2 tubular cells line cultured with high glucose (HG) showed exacerbated lipid deposition. The effect was partly due to increased CD36 expression via the AKT-PPARγ signaling pathway. This underlines/emphasizes the relevance of FA or cholesterol uptake in intracellular lipid accumulation [55,56]. Furthermore, CD36 is involved in HG-induced EMT in renal tubular epithelial cells [57]. This is of great interest because we also observed EMT-like changes in the kidneys in our db/db mouse model; for example, the expression of the transcription factor Snail1 was increased and the tubular epithelial marker E-cadherin was reduced [41]. Although hyperglycemia is a clinical hallmark in T1DM and EMT-like changes could already be detected in the STZ model [58], but in our mice with a T1DM-like phenotype, both the HG-CD36 and the CD36-EMT axis do not play a major role.

In the T2DM model, however, a reduction of MORG1 expression inhibits both TGF-β1 and EMT-like changes [41], conforming the CD36 expression pattern shown here in heterozygous MORG1 mice (Figure 3B).

In line with the increased FAS in non-diabetic MORG1 knockout mice, the CD36 protein is also highly expressed when MORG1 is reduced (Figure 3A,B). This is intriguing, as the uptake of extracellular FA and triacylglycerol synthesis are promoted by transcription factor PPARγ, which is directly activated by HIF1 [21].

Consistent with CD36 expression in the STZ-induced T1DM model, there is also a significant diabetes*MORG1 genotype interaction in T2DM, but this results from different and partially opposing effects. However, in mice with the single-allele deficiency of MORG1, there is significantly lower renal CD36 expression compared to non-diabetic kidneys, independent of diabetes type.

It is believed that lipotoxicity in patients with DN can mechanistically results from cell-specific stress (e.g., CD36-mediated cellular stress in tubular cells), as well as from generic cellular stress (e.g., altered mitochondrial energy production) [59]. Mitochondrial dysfunction is a well-recognized pathologic feature which triggers fibrosis [60]. Mitochondrially encoded cytochrome c oxidase subunit 1 (MTCO1) is one of the core subunits of complex IV (the final enzyme of electron transport chain of mitochondrial oxidative phosphorylation) and can be used as a mitochondrial marker. Recently, Haraguchi et al. showed an accumulation of oxidative modified mitochondria in the damaged diabetic proximal renal tubules after 10 weeks of STZ-induced T1DM in C57BL/6J mice [61]. We also selected MTCO1 as a mitochondrial target, but could not confirm diabetes-induced accumulation of mitochondria in STZ-T1DM (Figure 3C). In contrast to the model of Haraguchi et al., our mice have a different genetic background (namely, C57BLKS and not C57BL/6J) and administered with a lower dose of STZ (50 mg/kg body weight for 4 consecutive days vs. 70 mg/kg body weight for 5 consecutive days) [61].

Similarly, in our T2DM model, we did not detect any diabetic effect on MTCO1 levels (Figure 3D). Interestingly, kidneys with reduced MORG1 expression (MORG1 heterozygous) showed significantly intense MTCO1 staining (Figure 3D). We interpret higher levels of MTCO1 with increased mitochondrial fission. Mitochondrial fission generates new organelles, facilitates quality control and is a mechanism for preconditioning to be prepared for metabolic stress [62]. Various studies have demonstrated that hypoxia-preconditioning, pharmacologic and/or genetic activation of HIF protects from kidney injury [37,40,41,63,64,65]. Considering the fact that mitochondrial fission is regulated via ERK1/2 signaling [66], and, at low concentrations, MORG1 also enhances ERK activation [32]; this novel finding confirms that the MORG1 heterozygous mice are protected because of preconditioning. Supporting our hypothesis, it has been shown that increased expression of the key transcriptional regulator of mitochondrial biogenesis, PGC-1α, in renal tubule cells protects against chronic kidney disease [67].

### 3.3. Single-Allele Deficiency of MORG1 Restores Pathological Changes in Renal FAO

Tubular cells have a high basal level of energy demand, which is mostly produced by the β-oxidation of FA, due to greater ATP yield compared to oxidation of only glucose [16,18,68]. Decreased FAO in these renal epithelial cells has been reported to play a particular role in kidney fibrosis: on the one hand, the inhibition of FAO leads to a fibrotic phenotype, and, on the other hand, restoring FA metabolism protected mice from TIF [16].

**Figure 4:**
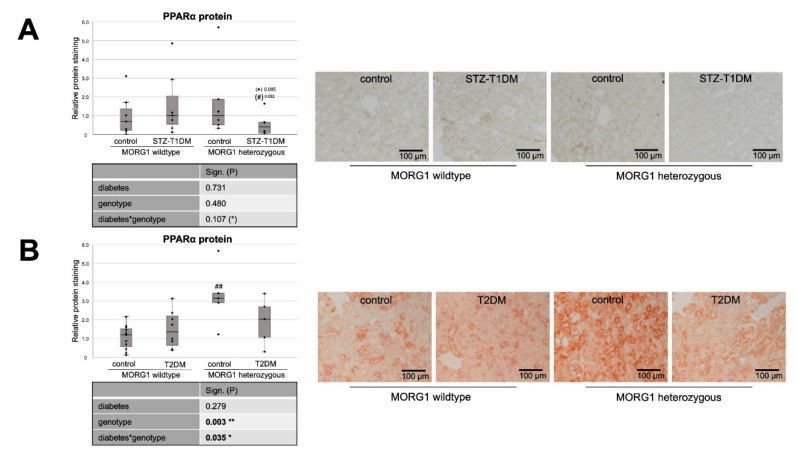
Role of MORG1 in renal expression of transcriptional factor of β-oxidation. (**A**,**B**) Immunohistochemistry of PPARα (peroxisome proliferator activated receptor alpha) protein in STZ-T1DM and T2DM with 10 non-overlapping images per animal and semi-quantitative analysis of the staining. Representative images are shown next to the graphs (magnification ×200). The significances shown in the tables were assessed by two-way ANOVA with diabetes (control or STZ-T1DM/T2DM) and genotype (MORG1 wild-type or MORG1 heterozygous) as the two factors. Diabetes, genotype: main effect of diabetes/genotype, respectively. Diabetes*genotype: interaction. Bold: statistically significant. (*) Biologically relevant. Significances shown next to each bar was assessed by using Mann–Whitney U test; * *p* ≤ 0.05 in between same genotype; ## *p* ≤ 0.01 in between different genotypes; (*) biological relevant in between same genotype; (#) biological relevant in between different genotypes.

**Figure 5:**
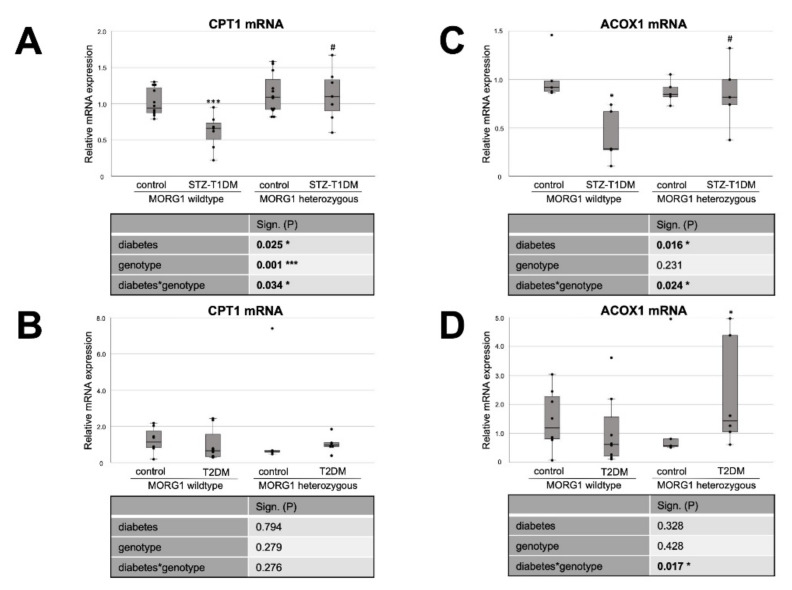
MORG1 effect on expression of key enzymes of FAO (fatty acid oxidation) in diabetic kidneys. (**A**,**B**) Real-time analysis of CPT1 (carnitine palmitoyl transferase 1) mRNA expression in STZ-T1DM and T2DM. (**C**,**D**) Real-time analysis of ACOX1 (Acyl-CoA Oxidase 1) mRNA expression in STZ-T1DM and T2DM. (**A**–**D**) The significances shown in the tables were assessed by two-way ANOVA with diabetes (control or STZ-T1DM/T2DM) and genotype (MORG1 wild-type or MORG1 heterozygous) as the two factors. Diabetes, genotype: main effect of diabetes/genotype, respectively. Diabetes*genotype: interaction. Bold: statistically significant. Significances shown next to each bar was assessed by using Mann–Whitney U test; * *p* ≤ 0.05 in between same genotype; *** *p* ≤ 0.001 in between same genotype; # *p* ≤ 0.05 in between different genotypes.

Therefore, we investigated the expression of key enzymes of FAO pathways: peroxisome proliferator activated receptor alpha (PPARα), carnitine palmitoyltransferase 1 (CPT1) and acyl-CoA oxidase (ACOX1) (Figure 4 and Figure 5). PPARα is the key transcriptional factor that directly controls genes involved in β-oxidation, FA uptake and triglyceride catabolism [68]. FAs undergo transport into mitochondria, where FAO takes place, by CPT1 and degradation (β-oxidation) by ACOX1 [20,21].

Whereas our wild-type animals, in contrast to the literature, showed no changes in tubular PPARα protein expression in either STZ-T1DM or T2DM (Figure 4), there is the expected diabetic effect (in this study: significant in STZ-T1DM and tangential in T2DM) in the expression of CPT1 and ACOX1 (Figure 5). The diabetes-induced decrease in both FAO markers is abolished in mice with single-allele deficiency of MORG1, which translates into a significant diabetes*genotype interaction in STZ-T1DM (for CPT1 and ACOX1) and T2DM (for ACOX1). One possible explanation as to why there is no detectable change in expression of PPARα while its target genes CPT1 and ACOX1 are reduced is that TGF-β1 influences FAO. TGF-β1, which is upregulated in both T1DM and T2DM, can decrease FAO by classical signaling (via Smad3 and ERK1/2 pathway) by reducing PPARα and its target genes CPT1 and ACOX1; additionally, it can also inhibit PGC-1α and PPAR at epigenetic levels [16,67].

Interestingly, in non-diabetic animals with reduced MORG1 expression PPARα is increased. Because this is consistent with the expression patterns for FAS and CD36 (compare Figure 4B with Figure 1B and Figure 3B and Figure 4A with Figure 1A and Figure 3A), we assume a feedback mechanism here to maintain physiological lipid metabolism. Correlation analysis of non-diabetic control animals revealed a strong positive correlation (ρ 0.74**) between PPARα and FAS in the T2DM model (compare Figure 4B with Figure 1B).

In the presence of diabetes and lower MORG1 expression, PPARα values return to basal levels. There is a biological relevance in STZ diabetes (Figure 4A) and even a significant interaction between diabetes and MORG1 genotype in T2DM (Figure 4B). This may be related to increased HIF2α expression/stabilization in these animals, as it was shown in the fatty liver that HIF2α-mediated activation of ERK decreases PPARα activity [69]. Previous studies in the db/db model showed that diabetic animals with single-allele deficiency of MORG1 had significantly higher HIF2α levels [41], which was also confirmed in the STZ-T1DM model (data not shown).

### 3.4. Reduction of MORG1 Ameliorates Renal Fibrosis and Lipid Accumulation

**Figure 6:**
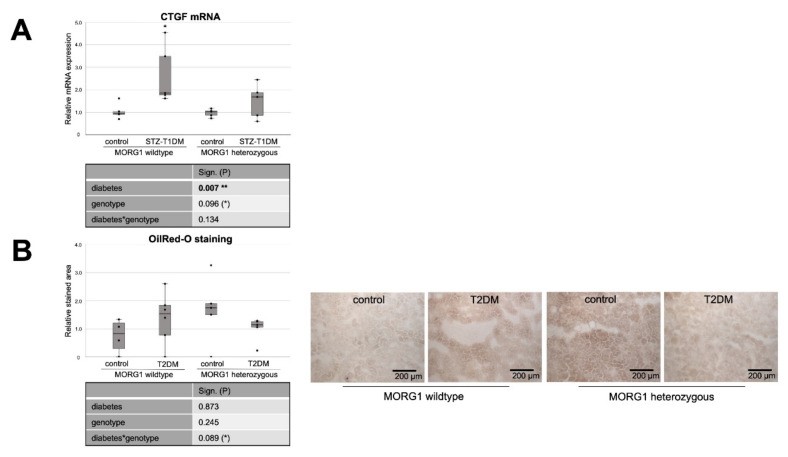

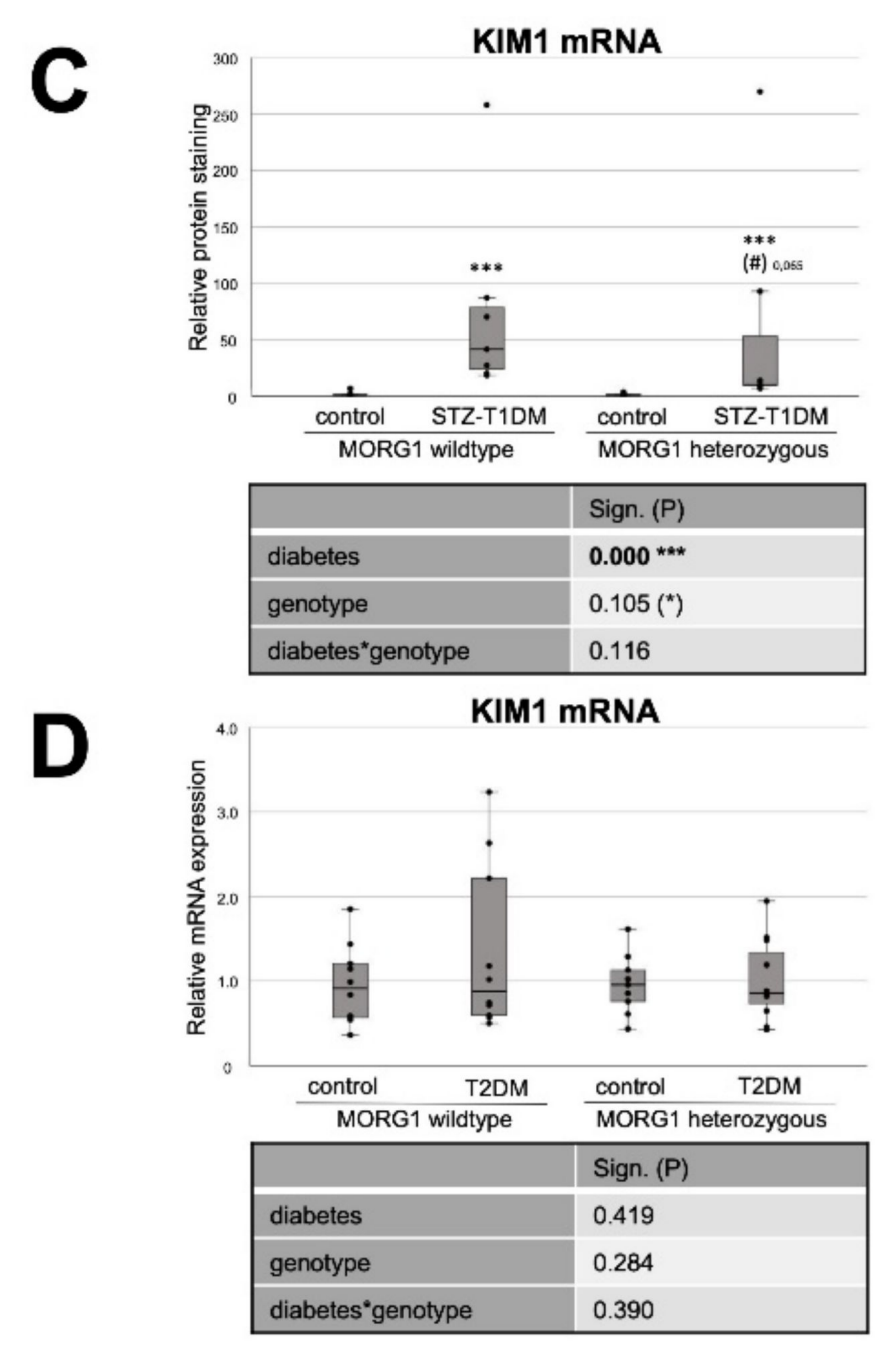
Effect of diabetes and MORG1 on TIF and lipid accumulation. (**A**) Real-time analysis of CTGF (connective tissue growth factor) mRNA expression in STZ-T1DM. (**B**) Quantitative analysis of OilRedO staining of kidney sections from T2DM animals (graph) and representative images next to the graph (magnification ×100). (**C**,**D**) Real-time analysis of KIM1 (kidney injury molecule-1) mRNA expression in STZ-T1DM and T2DM (**A**–**D**) The significances shown in the tables were assessed by two-way ANOVA with diabetes (control or STZ-T1DM/T2DM) and genotype (MORG1 wild-type or MORG1 heterozygous) as the two factors. Diabetes, genotype: main effect of diabetes/genotype, respectively. Diabetes*genotype: interaction. Bold: statistically significant. (*) Biologically relevant. Significances shown next to each bar were assessed by using Mann–Whitney U test; * *p* ≤ 0.05 in between same genotype; *** *p* ≤ 0.001 in between same genotype; (#) biological relevant in between different genotypes.

It has been reported that altered lipid metabolism, especially defective FAO, can promote TIF [16]. In addition, gene-expression analyses of a large number of human renal tubule samples showed that genes with metabolic functions represent the largest group of dysregulated genes in renal fibrosis [67]. Therefore, we investigated the expression of fibrosis-promoting factors CTGF and kidney injury molecule 1 (KIM1) (Figure 6). For KIM1, a type I membrane protein, a high induction of expression after acute injury and in fibrotic kidneys has been shown [70,71]. KIM1 is an early biomarker of AKI and has furthermore a potential role in predicting the long-term renal outcome [71]. It has also been shown that KIM1 expression correlates with proinflammatory chemokine MCP1 expression [70].

In the STZ-T1DM model, a clear and significant diabetes effect was detected on both CTGF and KIM1 mRNA expression (Figure 6A,C). However, diabetic conditions do not have such a strong effect on fibrosis marker expression when MORG1 is reduced. There is no statistical significance in heterozygous genotype between diabetic and non-diabetic animals (in the case of CTGF; Figure 6A). However, a direct group comparison of diabetic heterozygous animals with those of the MORG1 wild type shows a decrease with biological relevance (in case of KIM1; Figure 6C). Correlation analyses showed moderately significant inverse correlations between renal fibrosis and FAO (CTGF mRNA vs. CPT1 mRNA ρ—0.6**; CTGF mRNA vs. ACOX1 mRNA ρ—0.7***). In addition, a fair to moderately significant inverse correlation between damaged tubules and FAO (KIM1 mRNA vs. CPT1 mRNA ρ—0.4**; KIM1 mRNA vs. ACOX1 mRNA ρ—0.7***) was demonstrated.

We have already examined TIF in the T2DM animals with different MORG1 genotypes in our previous study [41]. The results obtained in a previous study correlate with the current findings for STZ-T1DM. Diabetes-induced CTGF mRNA and protein expression, as well as collagen I and fibronectin accumulation in the tubulointerstitium of the *db/db* mice, were less pronounced when a heterozygous MORG1 knockout genotype was present in addition to diabetes [41]. KIM1 expression analysis in the T2DM model showed no significance among the mice studied here, but a trend showing the same pattern as the other fibrosis markers from the previous study (Figure 6D and Reference [41]).

Our analyses of the expression of key proteins and enzymes of lipid metabolism in the kidneys of mice with T1DM-like phenotype and T2DM show clear evidence of alterations that may be causative for accumulation of fat in kidney tissue. To investigate the extent to which visible lipid droplets are already present, we applied Oil Red O staining. While no renal intracellular fat was detectable in the STZ mice (data not shown), lipid droplets could be visualized in type 2 diabetic kidneys (Figure 6C). This is consistent with the findings in kidney biopsies of patients with diagnosed type 2 DN [20]. In the background of the MORG1 heterozygous knockout, less lipid deposition was detectable in diabetic kidneys than in non-diabetic ones. However, a significance analysis across all groups indicates a statistical interaction between the T2DM and the MORG1 genotype that we consider to be biologically relevant, with *p* = 0.089 (Figure 6B). The results shown so far suggest that the accumulation of lipid droplets in db/db mice is not due to the increased synthesis of FA, and it is also less because of downregulated FAO. Rather, in this model, there is an oversupply of circulating lipids due to obesity per se that may be transported via CD36 into the kidney cells. Hence, the correlation analysis reveals a moderate positive correlation between tubular lipid accumulation and lipid uptake (OilRedO vs. CD36 protein ρ 0.6**).

As discussed above, we hypothesize a feedback mechanism in response to increased FA levels as an explanation for the elevated PPARα expression in non-diabetic heterozygous animals (see Figure 4). Oil Red O staining confirms increased lipid accumulation in these animals, to which the cells apparently respond by increasing PPARα. The efficacy of thereby decreasing renal lipid accumulation was demonstrated by pharmacological activation of PPARα by, for example, fenofibrate [72].

Evidence suggests that renal lipid accumulation may be the consequence, as well as the cause, of diabetic kidney injury [73]. In kidneys that show increased intracellular lipid droplets, β-oxidation pathways were downregulated [20]. Although we found a marked downregulation of the CPT1 and ACOX1 genes in STZ-T1DM, no lipid accumulation could be visualized. However, because the expression of fibrosis markers was already upregulated, it can be assumed that, in the STZ-T1DM model, changes in lipid metabolism are the consequence rather than the cause of early damage. In obesity-associated T2DM, it seems to be the other way around: lipid droplets are clearly visible, whereas fibrotic changes are still comparatively minor.

Of great interest in this context is a very recent study with the finding that overexpression of CPT1 in renal tubule cells protects against renal injury and fibrosis by restoring metabolic gain of function in FAO and mitochondrial homeostasis [74]. This is a promising finding, especially because overexpression of CPT1 attenuated the fibrotic phenotype even after FA-induced injury [74]. One negative aspect of this study, according to Reidy and Ross [75], is that all three employed murine models of renal fibrosis have well-known limitations regarding their relevance to human chronic kidney disease.

## 4. Conclusions

In this animal-based study, key markers of de novo FA/cholesterol synthesis, lipid uptake and FAO were examined in parallel for both major diabetes types and related to TIF and lipid accumulation. Alterations in lipid metabolism are present in both STZ-T1DM and T2DM, but therapeutic targets on lipid metabolism, such as PPAR agonists or CD36 antagonists, to prevent diabetes-induced TIF should be adapted to the diabetes type. Another and possibly better target is MORG1, which appears to play different roles in lipid metabolism, depending on the underlying diabetes type. Although our current study has not yet fully established a causal link between MORG1 expression and the observed changes in gene expression levels, the results, in agreement with several previous observations, support a potential implication of MORG1 in pathogenesis of lipid-associated kidney fibrosis. In other words, reducing MORG1 levels restores physiological lipid metabolism and is renoprotective, regardless of the diabetes type.

## Figures and Tables

**Table 1:** List of primer pairs and annealing temperatures.

Gene	Sense and Antisense Primers	T_ann._
ACOX1	5′-GCCCAACTGTGACTTCCATC-3′ 5′-GCCAGGACTATCGCATGATT-3′	60 °C [44]
CPT1	5′-TCCATGCATACCAAAGTGGA-3′ 5′-TGGTAGGAGAGCAGCACCTT-3′	60 °C [45]
CTGF	5′-TGCTGTGCAGGTGATAAAGC-3′ 5′-AAGGCCATTTGTTCACCAAC-3′	58 °C [46]
FAS	5′-CCTGGATAGCATTCCGAACCT-3′ 5′-ACACATCTCGAAGGCTACACA-3′	61 °C [47]
HMG-CoA-Red	5′-AGCCGAAGCAGCACATGAT-3′ 5′-CTTGTGGAATGCCTTGTGATTG-3′	57 °C [48]
HPRT1	5′-TGGATACAGGCCAGACTTTGTT-3′ 5′-CAGATTCAACTTGCGCTCATC-3′	59 °C [49]
KIM1	5′-ATGAATCAGATTCAAGTCTTC-3′ 5′-TCTGGTTTGTGAGTCCATGTG-3′	58 °C [50]
SREBP2	5′-CAAGTCTGGCGTTCTGAGGAA-3′ 5′-ATGTTCTCCTGGCGAAGCT-3′	61 °C [51]

ACOX1 = Acyl-CoA Oxidase 1, CPT1 = carnitine palmitoyltransferase 1, CTGF = connective tissue growth factor, FAS = fatty acid synthase, HMG-CoA-Red = 3-hydroxy-3-methylglutaryl-Coenzyme A reductase, HPRT1 = hypoxanthine phosphoribosyl transferase 1, KIM1 = kidney injury molecule-1, SREBP2 = sterol regulatory element-binding protein 2, T_ann_. = annealing temperature.

## Data Availability

The data presented in this study are available on request from the corresponding author.

## Supplementary Materials

The following are available online at https://www.mdpi.com/article/10.3390/biomedicines10010030/s1. Method S1: MACRO used for FAS-staining, exemplary for all IHC evaluations. Figure S1: MORG1 protein expression in STZ-T1DM model.

## Abbreviations

ACC, acetyl-CoA carboxylase; ACOX1, acyl-CoA oxidase; AGE, advanced glycation end-products; ANG II, angiotensin II; BSA, bovine serum albumin; CD36, cluster of differentiation 36; CPT1, carnitine palmitoyltransferase 1; CTGF, connective tissue growth factor; DAB, diabminobenzidine; DAPI, 4′,6-diamidino-2-phenylindole; DN, diabetic nephropathy; ECM, extracellular matrix; EMT, epithelial-to-mesenchymal transition; EPO, erythropoietin; ERK1/2, extracellular signal-regulated kinase; ESRD, end-stage renal disease; FA, fatty acid; FAO, fatty acid oxidation; FAS, fatty acid synthase; HDL, high density lipoprotein; HIF1/2, hypoxia-inducible factor; HMG-CoA-Red, 3-hydroxy-3-methylglutaryl-Coenzyme A reductase; HPRT1, hypoxanthine phospho-ribosyltransferase 1; KIM1, kidney injury molecule-1; MAPK, mitogen-activated protein kinase; MCP1, monocyte chemoattractantprotein-1; MORG1, mitogen-activated protein kinase organizer 1; MP1, MEK partner 1; MTCO1,mitochondrially encoded cytochrome C oxidase I; PGC-1α, peroxisome proliferator-activated receptor gamma coactivator 1-alpha; PHD3, prolyl hydroxylase domain protein 3; PPARα, peroxisome proliferator activated receptor alpha; SCD1, stearoyl-CoA desaturase 1; RAF, rapidly accelerated fibrosarcoma; SREBP2, sterol regulatory element binding proteins; STZ, streptozotocin; T1DM, type 1 diabetes mellitus; T2DM, type 2 diabetes mellitus; TGF-β1, transforming growth factor β1; TIF, tubulointerstitial fibrosis.
