# Supplementary material for: MORG1—A Negative Modulator of Renal Lipid Metabolism in Murine Diabetes
Source: Biomedicines. 2021 Dec 23;10(1):30. doi: 10.3390/biomedicines10010030 (PMC8772719; doi:10.3390/biomedicines10010030)
Supplement: Supplementary file 1 [file biomedicines-10-00030-s001.zip › biomedicines-1504965-supplementary.pdf]

biomedicines

Supplementary Material:

Method S1: *MACRO used for FAS-staining, exemplary for all IHC evaluations.*

```
dir1 = getDirectory("Choose Source Directory ");
setBatchMode(true);


dir2=getFileList(dir1);
    rowNumber=0;
for (jj=0;jj<dir2.length;jj++){


if (!endsWith(dir2[jj],"/"))
    continue;


list = getFileList(dir1+dir2[jj]);


for (ii=0; ii<list.length; ii++) {
showProgress(ii+1, list.length);


   // INSERT MACRO HERE


open(dir1+dir2[jj]+list[ii]);
//run("Duplicate...", "title=working_copy.tif");
//selectWindow(list[i]);
//run("Color Threshold...");
// Color Thresholder 2.1.0/1.53c
// Autogenerated macro, single images only!
min=newArray(3);
```

```
max=newArray(3);
filter=newArray(3);
a=getTitle();
run("HSB Stack");
run("Convert Stack to Images");
selectWindow("Hue");
rename("0");
selectWindow("Saturation");
rename("1");
selectWindow("Brightness");
rename("2");
min[0]=0;
max[0]=255;
filter[0]="pass";
min[1]=22;
max[1]=63;
filter[1]="pass";
min[2]=0;
max[2]=255;
filter[2]="pass";
for (i=0;i<3;i++){
  selectWindow(""+i);
  setThreshold(min[i], max[i]);
  run("Convert to Mask");
  if (filter[i]=="stop")    run("Invert");
}
imageCalculator("AND create", "0","1");
```

```
imageCalculator("AND create", "Result of 0","2");
for (i=0;i<3;i++){
  selectWindow(""+i);
  close();
}
selectWindow("Result of 0");
close();
selectWindow("Result of Result of 0");
rename(a);
// Colour Thresholding------------
run("Create Selection");
roiManager("Add");


close();
open(dir1+dir2[jj]+list[ii]);
roiManager("Select", 0);
//selectWindow("working_copy.tif");
//roiManager("Select", 0);
run("Measure");


roiManager("Delete");
close();
// INSERT MACRO HERE
    //saveAs("TIFF", dir2+list[i]);
    if(ii==0)
      setResult("testreihe", rowNumber, dir2[jj]);
rowNumber++;
```

```
}

//Table.rename("Results", dir2[jj]);
```

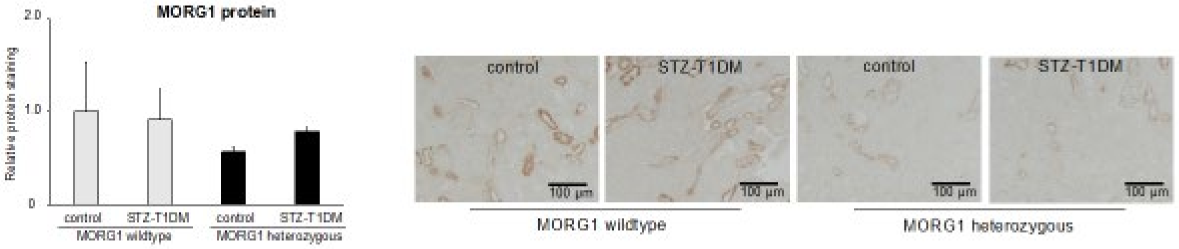

Figure S1: MORG1 protein expression in STZ-T1DM model. Immunohistochemistry of renal MORG1 protein in wildtype and heterozygous mice and semiquantitative analysis of the staining. Representative images are shown next to the graph (magnification x200).
